# A Brief Boot Camp for 4th-Year Medical Students Entering into Pediatric and Family Medicine Residencies

**DOI:** 10.7759/cureus.488

**Published:** 2016-02-09

**Authors:** Rebekah Burns, Mark Adler, Karen Mangold, Jennifer Trainor

**Affiliations:** 1 Pediatrics, Seattle Children's Hospital - University of Washington School of Medicine; 2 Pediatrics, Northwestern University Feinberg School of Medicine, Chicago, IL, USA

**Keywords:** boot camp, pediatrics, communication skills, clinical skills, medical student education

## Abstract

The transition from medical student to intern is a challenging process characterized by a steep learning curve. Focused courses targeting skills necessary for success as a resident have increased self-perceived preparedness, confidence, and medical knowledge. Our aim was to create a brief educational intervention for 4th-year medical students entering pediatric, family practice, and medicine/pediatric residencies to target skills necessary for an internship.

The curriculum used a combination of didactic presentations, small group discussions, role-playing, facilitated debriefing, and simulation-based education. Participants completed an objective structured clinical exam requiring synthesis and application of multiple boot camp elements before and after the elective. Participants completed anonymous surveys assessing self-perceived preparedness for an internship, overall and in regards to specific skills, before the elective and after the course. Participants were asked to provide feedback about the course.

Using checklists to assess performance, students showed an improvement in performing infant lumbar punctures (47.2% vs 77.0%; p < 0.01, 95% CI for the difference 0.2, 0.4%) and providing signout (2.5 vs. 3.9 (5-point scale) p < 0.01, 95% CI for the difference 0.6, 2.3). They did not show an improvement in communication with a parent. Participants demonstrated an increase in self-reported preparedness for all targeted skills, except for obtaining consults and interprofessional communication. There was no increase in reported overall preparedness. All participants agreed with the statements, “The facilitators presented the material in an effective manner,” “I took away ideas I plan to implement in internship,” and “I think all students should participate in a similar experience.” When asked to assess the usefulness of individual modules, all except order writing received a mean Likert score > 4.

A focused boot camp addressing key knowledge and skills required for pediatric-related residencies was well received and led to improved performance of targeted skills and increased self-reported preparedness in many targeted domains.

## Introduction

The transition from medical student to intern is a challenging process characterized by a steep learning curve as new doctors are suddenly faced with markedly increased clinical responsibilities. These changes require strong communication and organizational skills as well as basic, practical medical knowledge [1].

The final year of medical school within the United States often consists of minimal required content and maximum freedom to choose amongst elective courses and non-clinical pursuits [2]. A few medical schools have instituted brief, mandatory courses before graduation, focused on communication, procedural and life skills, and the management of common medical emergencies. After participation in a three-week capstone course at the University of California, San Francisco, students felt better prepared in several areas including identifying sick patients, obtaining help, communicating, carrying out daily patient-care responsibilities, and maintaining their own well-being [3]. Students who participated in a week-long elective “Internship Boot Camp” course at the Mayo Medical School, consisting of a mix of simulation and problem-based learning sessions, recalled it as the most important aspect of medical school in preparation for the intern year [4]. Senior electives directed at students entering the field of surgery have been shown to increase knowledge and improve confidence in a wide variety of skills including procedures, education, and communication [5-6]. Boot camp-style courses have been demonstrated to increase participant knowledge, skills, and confidence in many fields of medicine [7]. No such courses have been described for students entering pediatric-based residencies, however.

While there are many required skills that overlap with other fields of medicine, pediatric residents will have the specific need to perform age-specific procedures as well as communicate with children and their families. Both immersive, case-based simulation and task/procedural training are particularly useful training techniques for graduating medical students. These can expose participants to realistic challenges and let them experience the consequences of their decisions and actions while providing a safe environment without risk to actual patients. We hypothesize that a focused course addressing the key knowledge and skills required for residency will help to graduate medical students entering into pediatrics, medicine/pediatrics, and family medicine feel better prepared for internship and, ultimately, improve patient safety and satisfaction for those cared for by interns who have completed the course. We conducted a pilot study to evaluate the curricular content and delivery.

## Materials and methods

Fourth-year medical students at the Northwestern University’s Feinberg School of Medicine (FSOM) who matched into residencies in pediatrics, medicine/pediatrics, or family medicine were offered an ungraded, five-day elective entitled “Pediatric Internship Boot Camp” in the spring of 2013. The elective used a combination of brief didactic presentations, small group discussions, role playing, facilitated debriefing, and simulation-based education. This study was approved by the Institutional Review Board of the Ann and Robert H. Lurie Children's Hospital of Chicago (approval #2012-14872), and all participants provided written consent prior to participation.

### Curriculum

Day 1 and 2 modules were taught in small groups using cased-based role-play with four students per trained facilitator (Tables 1-2). The students were given four pediatric cases each morning for use during all modules during the day. On day 3, students rotated through skill sessions in groups of four students per two instructors (Table 3).

**Table 1:** Day 1 curriculum

Module	Targeted Skill	Content
Assessment and plan formulation	Medical knowledge	Students formulate assessments for their “patients” and proposed plans for further evaluation and care Present in both system-based and problem-based formats to their small groups
Task prioritization	Organization	Students make lists of concrete tasks that must be completed for their patients during a work day Small group discussion on the priorities for completion
Interprofessional communication (IPC)	Verbal communication	Brief lecture about IPC Three video vignettes demonstrating suboptimal IPC Brainstorm and role play strategies to improve communication Three vignettes are re-presented demonstrating constructive IPC
Answering pages	Verbal communication	Brief lecture about answering pages Presentation of simulated pages regarding the patients Students role play answering pages for their respective patients
Signout	Verbal communication	Lecture about strategies for safe signout with focus on the I-PASS mnemonic [8], including examples Updated information on the patients given Students practice signing out their patients to one another

**Table 2:** Day 2 curriculum

Module	Targeted Skill	Content
Communicating with consultants	Verbal communication	Brief lecture about requesting consultation Students role-play asking for a question-focused consult for their “patients.”
Delivering difficult news	Verbal communication	Lecture about SPIKES protocol for delivering difficult news[9] Students view video examples of delivering difficult news and discuss strategies to improve communication After being provided with the recommendations from their “consultants,” they role-play giving difficult news
Order writing	Written communication	Students complete admission orders for two of their “patients"
Prescription writing	Written communication	Brief lecture about how to write prescriptions, including examples Students practice writing prescriptions for the “patients”

**Table 3:** Day 3 curriculum

Module	Targeted Skill	Content
ABCs	Clinical skills	Brief lectures about assessment, basic airway support, bag-mask ventilation, and chest compressions Drill-like deliberate practice of skills using pediatric simulators
Neonatal intubation	Clinical skills	Brief lecture about the anatomy of the neonatal airway and intubation Hands-on practice using task trainers
Defibrillation	Clinical skills	Lecture about defibrillation Students allowed time to manipulate defibrillator and become comfortable with its use Participation in simulations requiring defibrillation and cardioversion
Infant lumbar puncture (LP)	Clinical skills	Video demonstrating performance and proper technique for infant LP Baseline assessment performed using lumbar puncture checklist (LPC) that includes yes/no assessment of 15 items Students practice using high fidelity task trainers
Informed consent	Verbal communication	Lecture about informed consent with specific focus on LP Students practice obtaining informed consent

On day 4 of the course, students rotated through four immersive simulations in small groups. Each was followed by a debriefing session to discuss team dynamics, communication skills, and knowledge gaps around specific medical content. Simulations included a confederate “nurse” and “parent” to provide the history and a nursing student. An additional technician ran the simulations. After the debriefing period, groups were allowed to repeat the scenario to practice leadership, communication, and clinical skills. The four immersive simulations were:

*Septic Shock in a Child with Known Acute Lymphoblastic Leukemia (ALL): *A high-fidelity pediatric simulator was used to present the case of a child with ALL admitted to the oncology service for fever. Clinical goals included identifying septic shock, obtaining the appropriate infectious workup for fever and neutropenia, initiation of appropriate fluid resuscitation in a child with septic shock, and delivery of appropriate antimicrobial therapy.

*Respiratory Distress in an Infant with Bronchiolitis*: A high-fidelity infant simulator was used to present the case of an admitted infant with respiratory distress. The clinical goals included identification of respiratory distress, utilization of positioning and suctioning, and identification of different mechanisms for oxygen delivery and non-invasive positive pressure ventilation.

*Status Epilepticus in a Febrile Neonate:* A high-fidelity neonatal simulator was used to present the case on a febrile newborn in the emergency department in status epilepticus. Clinical goals included identifying status epilepticus, providing medical management for seizures, recognizing respiratory distress, providing mag-mask ventilation, and identifying the necessary evaluation of a febrile infant.

*Supraventricular Tachycardia (SVT) in a Non-Verbal Patient:* A high-fidelity pediatric simulator was used to present the case of a non-verbal child with fussiness and intolerance of feeds who was found to have SVT. Clinical goals included identification and management of stable SVT and anticipation and planning for the management of unstable SVT.

Self-reflection and peer feedback were encouraged and facilitated throughout the course. The final day of the elective was devoted to an individual objective structured clinical exam (OSCE) requiring students to use skills from the previous days to care for a patient throughout an emergency room visit. All students were provided with specific, detailed feedback at the end of the session.

### Evaluation

Pre-Boot Camp OSCE: 

In the week prior to the elective, students participated in an individual OSCE. The case required students to take a history from a facilitator acting as a standardized parent (SP), discuss the plan with the SP, and sign out the patient to another facilitator acting as a cross-covering resident. The SP completed the Gap-Kalamazoo Communication Skills Assessment (GKCSA) Peer/Facilitator form to assess communication. This instrument uses a five-point Likert scale (1=poor, 2=fair, 3=good, 4=very good, 5=excellent) to assess nine dimensions of communication (building a relationship, opening the discussion, gathering information, understanding the patient’s perspective, sharing information, reaching agreement on problems and plans, providing closure, demonstrating empathy, and communicating accurate information) [11]. The second rater completed the I-PASS Study Verbal Handoff Assessment (ISVHA) [8]. Because only one handoff was being observed, the tool was adapted into a yes/no checklist rather than a five-point Likert scale assessing frequency. 

Post-Boot Camp OSCE: 

The course culminated with an individual OSCE. The students were given a novel case that required the synthesis and application of boot camp elements including communication with a trained SP, performing an infant LP and signing the patient out to a cross-covering resident. During the OSCE, a facilitator observed the students interactions with the SP remotely and acted as the cross-covering resident. The facilitator completed the GKCSA Peer/Facilitator form, the SP the GKCSA Parent/Family form, and the student the GKCSA Self form. The handoff was again assessed using the modified ISVHA. Lumbar puncture (LP) performance was re-evaluated with the lumbar puncture checklist (LPC).

Preparedness Surveys: 

All students matching into the targeted residencies were asked to complete an anonymous on-line survey to assess their self-perceived preparedness for an internship. They were asked to rate their agreement with the statement “Overall, I feel well prepared for internship” as well as assess their preparedness for specific tasks, skills and challenges encountered in residency using a five-point Likert scale (1=strongly disagree, 2=disagree, 3=neutral, 4=agree, 5=strongly agree). Surveys were distributed in the month before the boot camp with an identical survey sent in the fourth month of internship. Boot camp participants were also asked to complete the survey a week after participating in the course. Unique, self-assigned study codes were used so that individuals’ responses could be compared across time points.

Student Feedback: 

At the end of each day, students were asked to assess the course using a five-point Likert scale (1=strongly disagree, 2= disagree, 3=neutral, 4=agree, 5=strongly agree). For days 1-3, students were asked to provide assessments of the usefulness of individual modules, the balance between didactics and interactive components, the ability of the facilitators to present the material effectively, and whether they would use what they had learned in an internship. On day 4, they were asked to assess the usefulness of the individual cases as well as whether they felt that team communication skills had been addressed. They were also encouraged to give specific feedback throughout. At the end of the week, they were asked about their opinion of the elective overall.

### Statistical analysis

Survey responses and OSCE data were analyzed using paired t-tests and repeated measure ANOVA. All tests used p < 0.05 as the significance level. Statistical analysis was performed using Stata (version 11.2, StataCorp, College Station, TX) and IBM SPSS (version 21, IBM Corporation, Armonk, NY). 

## Results

### Participation

Sixteen graduating medical students participated in the boot camp. Twelve were entering into categorical pediatric residencies (75%), three into family medicine (19%), and one into a combined internal medicine/pediatrics program (6%). Six were male. The students would become interns at 14 different institutions across the country with two remaining within our institution. Fifteen students participated in the post-boot camp OSCE (94%).

### Resources

The boot camp required four facilitators for days 1-3. For rotating immersive simulations, two facilitators and one simulation technician were used for each case. The OSCE was accomplished with five facilitators and two standardized parents. An additional coordinator was present each day.

Annual costs include medical equipment, including LP trays and needles, sterile gloves, and endotracheal tubes. We used one high-fidelity pediatric simulator, one adult pediatric simulator, and two infant simulators. Two LP task trainers and two infant airway task trainers were also used. Costs for the use of our simulation center were not included as the medical school supports this facility. Our experience is that similar facilities would charge between $250-400/hour for the use of a typical simulation center for each simulation space used for immersive events and perhaps less for task training or room use, although this can vary from site to site.

### OSCE results

For the 15 students participating in both pre- and post-intervention LP assessments, the mean pre-intervention LPC score on the checklist was 47.2% (SD 0.1), with a mean post-intervention score of 77.0% (SD 0.1) (p < 0.01; 95% CI for the difference 0.2, 0.4).

For the twelve students who participated in both the pre- and post-boot camp handoff assessments, the mean pre-intervention score on the modified ISVHA for completion of the five elements was 2.5 (SD 1.0). The mean post-intervention score was 3.9 (SD 1.1) (p < 0.01; 95% CI for the difference 0.6, 2.3)

There were no changes detected in facilitator GKCSA scores. On average, students scored high on the nine components of the GKCSA during the initial OSCE. During the post-intervention OSCE, there was no difference between facilitator, SP, and self-GKCSA scores for individual items, except for demonstrating empathy (facilitator 4.6 [SD 0.6], SP 4.0 [SD 0.4], and self 3.9 [SD 0.8; p = 0.02]).

### Survey responses

Fourteen boot camp participants (88%) completed both the pre-boot camp and immediately post-boot camp surveys. Thirteen participants (81%) completed the survey administered during their intern year. Eleven students (69%) completed all three surveys.

Participants reported an increase in self-perceived preparedness for many of the skills targeted and practiced during the course (Table 4). Regarding the targeted skills, students did not report an increase in preparedness for communication with non-physician health professionals or obtaining a consult from another provider. We also did not see a statistically significant increase in the feeling of overall preparedness (3.3 [SD 0.6] vs. 3.7 [SD 0.7], p = 0.05; 95% CI for difference 0.0, 0.9). 

**Table 4:** Pre- and post-boot camp survey responses *p < 0.05

I feel that as in intern I will be well prepared to:	Mean Likert Score Pre-Boot Camp (SD)	Mean Likert Score Post-Boot Camp (SD)	Mean Paired Difference (SD)	CI for Difference
Prioritize when faced with multiple/responsibilities.	3.4 (0.8)	3.9 (0.6 )	0.6 (0.8)	0.1, 1.0*
Keep clinical data well organized.	3.4 (0.8)	3.7 (0.8)	0.3 (0.8)	-0.2, 0.8
Teach medical students.	3.6 (0.9)	3.7 (0.8)	0.1 (0.7)	-0.2, 0.5
Provide medical students with constructive feedback	4.0 (0.8)	4.1 (0.3)	0.1 (0.8)	-0.4, 0.6
Provide high quality and thorough signout to another resident when going off service.	3.2 (0.7)	3.7 (0.7)	0.5 (0.9)	0.0, 1.0*
Provide high quality and thorough signout about my patients to a covering resident.	3.2 (0.7)	3.9 (0.8)	0.6 (0.9)	0.1, 1.2*
Receive signout to cross cover another trainee's patients.	3.3 (0.8)	3.9 (0.8)	0.6 (0.8)	0.1, 1.0*
Communicate with other non-physician health professionals (e.g. nurses, respiratory therapists, etc).	3.9 (0.6)	4.1 (0.7)	0.1 (0.9)	-0.4, 0.7
Provide information to and obtain a clinical plan from a consult service regarding my patient.	3.5 (0.7)	3.9 (0.6)	0.4 (1.0)	-0.2, 1.0
Manage clinical queries from the nursing staff over the phone.	2.9 (0.8)	3.6 (0.9)	0.8 (0.8)	0.3, 1.2*
Complete medical documentation in a thorough and timely fashion.	3.6 (0.8)	3.9 (0.9)	0.3 (0.6)	-0.1, 0.6
Write comprehensive and correct admission orders.	3.1 (0.8)	3.8 (0.8)	0.7 (1.1)	0.1, 1.3*
Write out prescriptions for medications.	2.9 (1.0)	3.9 (0.6)	1.0 (1.0)	0.4, 1.6*
Inquire about the code status of a pediatric patient.	2.6 (0.7)	3.4 (0.7)	0.7 (0.9)	0.2, 1.2*
Deliver news to the family of a patient, such as the new diagnosis of a malignancy.	2.9 (0.9)	3.4 (0.6)	0.5 (0.8)	0.1, 0.9*
Have a conversation about the concern for possible child abuse.	2.4 (0.8)	3.3 (0.7)	0.9 (0.8)	0.5, 1.4*
Obtain informed consent for a medical procedure.	3.6 (0.6)	4.4 (0.5)	0.7 (0.6)	0.4, 1.1*
Perform a lumbar puncture on an infant.	1.6 (0.7)	3.6 (1.0)	2.0 (1.0)	1.4, 2.6*
Properly use a defibrillator on a pediatric patient with a shockable dysrhythmia.	1.8 (1.0)	3.7 (0.8)	1.9 (0.9)	1.4, 2.5*
Correctly perform chest compressions on a pediatric patient.	2.8 (1.2)	4.3 (0.5)	1.5 (1.2)	0.8, 2.2*
Provide appropriate bag-mask ventilation to a pediatric patient.	2.5 (1.1)	4.2 (0.4)	1.7 (1.1)	1.1, 2.3*
Work with sick and dying children and their families.	3.4 (1.0)	3.9 (0.5)	0.5 (0.9)	0, 1.0*
Handle the consequences of making a medical mistake.	2.9 (0.9)	3.1 (0.7)	0.2 (0.7)	-0.2, 0.6
Manage work-life balance.	3.1 (0.7)	3.5 (0.5)	0.4 (0.8)	-0.1, 0.8

### Student feedback

Students overwhelming expressed satisfaction with the individual modules as well as the boot camp as a whole (Tables 5-6). The mean Likert score for the statement, “The module on (each individual module) was useful,” exceeded 4.0 (4.1-4.9, SD 0.3-1.0) for all modules in days 1-3, except for order writing (3.9, SD 0.8). Fifty-eight percent of the modules had a mean score > 4.5. The participants reported that all simulation-based sessions were useful with mean Likert scores of 4.8-4.9 (SD 0.2-0.4). The also reported that they learned about the presentation and medical management of the illnesses (4.7-4.8, SD 0.4-0.6) and team communication skills (4.8-4.9, SD 0.3-0.4) during these modules. In their free-text comments, the most common themes regarding the best aspect of the course included role-playing and skill practice.

**Table 5:** Daily feedback responses

Statement	Mean Likert Score (SD)			
	Day 1	Day 2	Day 3	Day 4
There was a good overall balance between the didactic component and interactive portion of the modules:	4.5 (0.6)	4.7 (0.5)	5.0 (0)	5.0 (0)
The facilitators presented the material in an effective manner:	4.6 (0.5)	4.9 (0.3)	5.0 (0)	5.0 (0)
I took away ideas that I plan to implement in internship:	4.8 (0.4)	5.0 (0)	5.0 (0)	5.0 (0)

**Table 6:** End of course feedback

Statement	Mean Likert Score	SD
I think that the assessment reflected the material in the course.	4.9	0.34
I think the end of assessment feedback was useful.	5.0	0.00
The facilitators presented the material in an effective manner.	4.9	0.25
I took away ideas that I plan to implement in internship.	4.9	0.25
I think all students should participate in a similar experience.	4.9	0.25

## Discussion

In Europe, it has been reported that first-year house officers feel unprepared for internship, specifically in basic clinical skills like prescribing, treatment, decision making, and emergency care [12-13]. Residency program directors within the United States report that interns commonly struggle with self-reflection and improvement, organization, application of knowledge, responsibility, and reliability. They feel these skills and attributes, along with communication, should be emphasized in the fourth-year medical school curriculum [14].

There are no reports of boot camps designed specifically for medical students entering into pediatric-based residencies. Using a combination of standardized patients, low-fidelity models, task trainers, and immersive simulations, we allowed students to practice a variety of skills before they are required to use them with actual patients, families, and other medical providers. We found our model to be feasible, both economically and resource-wise, within our institution. 

Participants demonstrated significant improvements in infant LP performance as assessed by the LPC. This supports previous evidence that skill improves after an educational intervention, but the translation to actual clinical care by our students is not known at this time [15]. Research evaluating residents’ ability to perform LPs successfully on infants after various educational interventions has been mixed [16-17]. 

Participants also demonstrated improved adherence to the I-PASS handoff tool after the course, as assessed by the modified ISVHA. Previous studies have shown that medical students adhere to handoff guidelines, both immediately after a targeted educational intervention as well as during future clinical rotations [18]. A multi-centered investigation has demonstrated a decrease in preventable medical errors after widespread implementation of the I-PASS tool [19]. Participants in our course may work in locations that use a variety of handoff tools that may or may not be standardized. Our hope is that by learning a commonly accepted tool during the boot camp, they will, at least, have an understanding of the importance of a safe and complete handoff and knowledge of common key components.

We did not see an improvement in communication using the GKCSA. Student scores during the pre-course OSCE were overall high. The lack of improvement likely demonstrates a ceiling effect on this portion of the assessment, as students were highly rated in their pre-intervention assessments. Additionally, the tool may not have the sensitivity to evaluate for subtle changes in regards to communication about difficult topics, such as the need for invasive procedures.

Participants subjectively reported feeling more prepared to perform tasks related to the skills taught during the elective with the exception of obtaining consults and interprofessional communication. We feel that this change was due to the curriculum as we did not see an increase in Likert scores addressing material not specifically addressed during the boot camp (teaching medical students, giving medical students feedback, documentation, working with sick children, handling the consequences of making a medical error, and managing work-life balance). Interestingly, we did not see a significant increase in overall self-reported preparedness suggesting there are other skills or knowledge that students may feel apprehensive about.

The students demonstrated a high level of engagement in the course and felt that the individual modules, as well as the course as a whole, were a valuable experience.

Limitations of this study include the inability to follow long-term outcomes related to intern performance. Because students moved to various institutions, it was not possible for us to compare them to peers in an unbiased manner. Although we attempted to contact all graduates entering into the targeted residencies to participate in the follow-up survey, we had a very poor response from people who did not participate in the boot camp. We were, therefore, unable to compare self-reported preparedness between students who did and did not participate in the boot camp during their intern year. Given the elective nature of the course, we had a small sample size of participants. There is also likely selection bias given that the students self-selected to participate. All evaluations were unblinded and performed by instructors who had been working with students throughout the course, which may have introduced bias into the evaluation process. Finally, the assessment tools, including the LPC, ISVHA, and GKCSA, have all been used to evaluate medical students and/or residents in other contexts but were not specifically validated in our population.

Future directions include continuing to revise the curriculum based on student feedback and assessment outcomes. We would like to continue to gather data from graduated participants of the boot camp and their program directors in order to continue to focus and develop the elective.

## Conclusions

A brief boot camp-style course for graduating medical students entering into pediatric or family medicine internships may lead to improved self-confidence and skills. Time constraints may be less severe at the end of medical school compared to the beginning of an internship, making this an ideal time to engage students in preparatory, hands-on learning prior to transitioning roles.
